# National trends in the prevalence and recurrence of anaphylaxis across all ages: The role of neighborhood deprivation and comorbidity (2002–2019)

**DOI:** 10.1016/j.waojou.2024.101005

**Published:** 2024-12-02

**Authors:** Ju Hee Kim, Eun Kyo Ha, Jeewon Shin, Nahyun Lee, Bo Eun Han, Man Yong Han, Eun Lee

**Affiliations:** a Department of Pediatrics, Kyung Hee University Medical Center, Kyung Hee University School of Medicine, Seoul, South Korea; b Department of Pediatrics, Kangnam Sacred Heart Hospital, Seoul, South Korea; c Department of Pediatrics, CHA Bundang Medical Center, CHA University School of Medicine, Seongnam, South Korea; d Department of Pediatrics, Chonnam National University Hospital, Chonnam National University Medical School, Gwangju, South Korea

**Keywords:** Anaphylaxis, Area deprivation index, Epidemiology, Recurrent

## Abstract

**Background:**

Understanding the trends of anaphylaxis and risk factors associated with its recurrence is essential for the effective management and prevention of this condition.

**Objective:**

This study aimed to analyze the prevalence trends of anaphylaxis and identify risk factors for recurrence, with a focus on the influence of neighborhood deprivation and comorbidities, across all age groups.

**Methods:**

We conducted a retrospective administrative cohort study on anaphylaxis utilizing the National Health Insurance-National Sample Cohort (NHIS-NSC) database in Korea (2002–2019). Anaphylaxis was defined with ICD-10 codes for the diagnosis combined with prescription codes. The Neighborhood Deprivation Index was used to identify the risk of recurrent anaphylaxis. Trends in the annual prevalence and recurrence of anaphylaxis were assessed through joinpoint regression and Cox proportional hazard models.

**Results:**

Out of the 1,137,861 individuals studied, 37,012 (3.25%) cases of anaphylaxis were identified. Among these, 5783 individuals (15.6%) experienced a recurrence, half of them experiencing it within the first year after the initial episode. The highest incidence of anaphylaxis was observed in children and adolescents, followed by middle-aged adults. A rapid increase in anaphylaxis cases was observed from 2002 to 2006 (Annual Percentage Change [APC], 33.2), followed by a more gradual increase until 2013 (APC, 12.8), and a stable trend from 2013 to 2019 (APC, 0.61). Males and adult age groups exhibited an increased risk of recurrence. Living in an area with neighborhood deprivation and the presence of comorbid conditions were associated with increased recurrence risk.

**Conclusions:**

The increasing prevalence of anaphylaxis and its association with certain risk factors calls for targeted intervention. Addressing neighborhood deprivation and comorbid conditions may aid in reducing the recurrence and overall burden of anaphylaxis.

## Introduction

Families and individuals dealing with anaphylaxis, a potentially life-threatening allergic reaction, are facing increasing challenges in accessing appropriate care.^1^ Over the past few decades, there has been a global increase in the prevalence of anaphylaxis. Countries such as Denmark,^2^ Australia,^3^ Spain,^4^ Hong Kong,^5^ and the United States^6^ have all experienced notable increases. Considering its relatively low prevalence, conducting population-based epidemiological studies on anaphylaxis is vital. Given the relatively low prevalence of anaphylaxis, it is crucial to conduct population-based epidemiological studies to accurately determine its real-world prevalence by utilizing a validated definition within nationwide data.^1^

Anaphylaxis is often triggered by foods, medications, and venom.^1^ While recurrence rates vary significantly, ranging from 3% to 76%, the factors contributing to recurrence are not fully understood. Recurrent episodes of anaphylaxis not only place a significant psychological and medical burden on patients and their guardians but also highlight a gap in our current understanding and management of this condition.^7^, ^8^, ^9^ Previous studies have primarily focused on limited aspects, such as comorbidities like asthma and atopic dermatitis.^7^^,^^8^^,^^10^^,^^11^ However, to enhance clinical outcomes and prevent recurrence, it is imperative to explore a broader spectrum of risk factors, including age-related variations and the role of comorbidity in the recurrence of anaphylaxis.^12^ The role of socioeconomic factors, particularly neighborhood deprivation, in the recurrence of anaphylaxis has been less clear, owing to the multifaceted nature of these elements. Therefore, a deeper examination of how these socioeconomic factors affect recurrent anaphylaxis is crucial.

This study aimed to investigate the epidemiology of anaphylaxis among individuals of all age groups from 2002 to 2019, focusing on identifying the risk factors associated with recurrence. This involved examining comorbid conditions, as well as socioeconomic and neighborhood deprivation status, using a validated definition of anaphylaxis within a nationwide health insurance data system.

## Methods

### Study design and data source

**Fig. 1:**
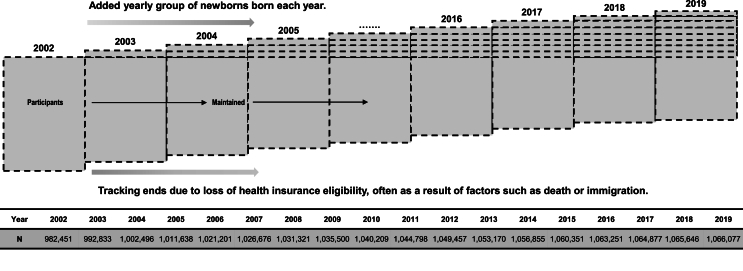
Schematic representation of the study population.

We conducted a retrospective administrative cohort study on anaphylaxis utilizing the National Health Insurance-National Sample Cohort (NHIS-NSC) database in Korea.^13^ This study spanned from January 1, 2002, to December 31, 2019. The NHIS-NSC is a representative sample cohort of the Korean population, comprising 2.2% of all residents in Korea, selected through a systematic stratified sampling method based on sex, age groups, and insurance premium levels. The cohort was assembled in 2002, and underwent periodic revisions, including annual additions of newly born infants and exclusion due to factors such as mortality and termination of health insurance benefits until 2019 (Fig. 1). In addition to socio-demographic variables such as age, sex, residential area, and insurance premium, this comprehensive database encompassed clinical variables. These included hospital utilization records of diagnosis codes (International Classification of Diseases, 10th Revision [ICD-10] codes), drug prescriptions, and intervention procedures, including surgical procedures. The use of de-identified individual data for research purposes was authorized under the National Health Insurance Act. The study protocol was reviewed and approved by the institutional review board of the Korea National Institute for Bioethics Policy (P01-201603-21-005).

### Definitions

In our study, anaphylaxis was systematically defined as meeting at least 1 of 3 conditions: (1) emergency room visits or hospitalizations with diagnostic codes for food-related anaphylactic reactions, drug or vaccine-induced anaphylactic reactions, or anaphylactic shock; (2) outpatient visits documented with codes for acute bronchospasm, stridor, or hypotension, along with recorded interventions such as cardiopulmonary resuscitation or the prescription of epinephrine or diphenhydramine; and (3) emergency room visits or hospitalizations associated with codes for allergic urticaria, angioneurotic edema, or poisoning by adverse effect or overdose of medications, coupled with the prescription of epinephrine or diphenhydramine.^14^ This operational definition of anaphylaxis was established based on its previous validation, demonstrating a positive predictive value of 64% (95% confidence interval [CI], 58–70%).^14^ To identify recurrence, we considered two or more diagnoses of anaphylaxis as indicative of a recurrent event. The date of the initial diagnosis was marked as the index anaphylaxis event, forming the basis for tracking subsequent occurrences.

### Covariates, age, and economic status

In our study, all participants were divided into 4 distinct age groups to better understand anaphylaxis across different life stages. These groups comprised children and adolescents (0–19 years old), young adults (20–39 years old), middle-aged adults (40–59 years old), and elderly individuals (≥60 years old). Additionally, the season during which the anaphylaxis occurred was categorized into 4 periods: spring (March–May), summer (June–August), autumn (September–November), and winter (December–February). In Korea, health insurance premiums are determined by property and income levels. Therefore, we determined the participants' economic status based on their health insurance premiums, dividing into three categories: low (below the 25th percentile), medium (25th to below the 75th percentile), and high (75th percentile or higher).

### Assessment of neighborhood deprivation

The Neighborhood Deprivation Index is a composite metric designed to assess material and social deprivation in the participants’ residential areas at their enrollment. Thus, we utilized the Neighborhood Deprivation Index to investigate the effect of poverty and inequality within a region on prevalence and recurrence of anaphylaxis, which might reflect the potential impact of socioeconomic status on the medical systems and medical usage according to regions.^15^ This index includes various regional variables, such as the proportion of individuals living alone, women-headed households, households without a car, households residing in apartment dwellings, households below the minimum housing standard, individuals aged 30–64 years without a high school diploma, economically active heads of households aged 15–64 years employed in manual labor, individuals aged ≥65 years, and the proportion of separated, divorced, or widowed individuals among those aged ≥15 years. The data for this index was derived from the 2010 Korean census,^16^ and each variable was standardized using a z-score to ensure comparability across different regions. In our study, the median score was −0.27. We categorized the levels of neighborhood deprivation into 3 distinct tiers: low (values below the 1st quartile, −0.41), intermediate (values equal to or greater than the 1st quartile but less than the 3rd quartile, −0.09), and high (values equal to or greater than the 3rd quartile, −0.09). This categorization allowed for a detailed analysis of the impact of varying degrees of neighborhood deprivation on our study outcomes.

### Comorbidity assessment by age groups

In our study, the evaluation of comorbid conditions was stratified by age groups due to the differing comorbidity profiles in children and adults. For children, our analysis focused on identifying risk factors associated with the most common allergic diseases. We meticulously assessed comorbidities of allergic diseases, including atopic dermatitis, allergic rhinitis, and asthma. Atopic dermatitis was defined as having one or more diagnoses of the condition, along with 2 or more prescriptions for topical corticosteroids. Allergic rhinitis was characterized by 5 or more diagnoses, and asthma was identified through either 2 or more diagnoses of asthma or at least 1 emergency room visit with an asthma diagnosis.^17^ For adults, in addition to allergic diseases, we evaluated the effects of other comorbidities using the Charlson Comorbidity Index (CCI), a widely recognized measure of chronic diseases for predicting long-term mortality.^18^ The CCI score was categorized into three levels: 0, no comorbidities; 1–2, a moderate level of comorbidities; and ≥3, a high level of comorbidities.

### Statistical analysis

In our study, the analysis was divided into 2 main components: investigating the changes in the prevalence of anaphylaxis over time and identifying the risk factors for recurrence.

To begin, we calculated the incidence rate of anaphylaxis per 10,000 person-years (PY), focusing on both the overall population and specific age groups. Covering the period from 2003 to 2019, we calculated the age-adjusted prevalence of anaphylaxis, using the population distribution from 2002 as a reference point. Our analysis utilized joinpoint regression to examine the annual prevalence trends from 2002 to 2019. This approach is essential for identifying significant shifts in trends, as it not only calculates the Annual Percentage Change (APC) and its CI but also determines the presence and nature of these trends. Additionally, the Average Annual Percent Change (AAPC) was computed for the entire period, providing a comprehensive measure of the average annual rate of change. We also conducted a stratified joinpoint regression analysis for subgroups defined by sex and age, ensuring that each annual prevalence estimate and subgroup AAPC for 2002–2019 was accompanied by the appropriate standard error (SE).

To assess the risk factors in individuals with recurrent anaphylaxis, we utilized the Cox proportional hazards model to calculate hazard ratios (HRs). We focused on potential variables such as sex, age, season, calendar year at the index anaphylaxis, neighborhood deprivation index, allergic comorbidities, and CCI score in adults. We determined the risk of recurrence by tracking both single episode and recurrent episodes within each subgroup, using the designated reference subgroup for the comparison of each characteristic. The follow-up period extended from the index anaphylaxis to the recurrence of anaphylaxis, death, or the end of the study, whichever came first. In our analyses, we adjusted for sex, age groups, and calendar year at index anaphylaxis, specifically excluding the independent variable from these adjustments. Additionally, we considered death as a competing risk to ensure more accurate HR estimates, thereby reflecting the true risk of anaphylaxis recurrence.

## Results

### Characteristics of study population

Our study encompassed a period from 2002 to 2019 and included a total of 1,137,861 individuals. Within this cohort, 37,012 (3.25%) were diagnosed with anaphylaxis, as detailed in Table 1. The distribution of sex among participants was approximately equal, with a nearly 1:1 male to female ratio. The median age at the time of the initial anaphylaxis episode was 37 years (interquartile range [IQR], 20–51 years). The majority of index anaphylaxis events occurred during the summer and autumn seasons, accounting for 29.2% and 28.3% of cases, respectively. Regarding comorbid conditions, 21.7% of those diagnosed with anaphylaxis had atopic dermatitis, 30.2% had allergic rhinitis, and 19.5% had asthma. Among the adult subgroup, 90.3% had a CCI score of 1–2, while 4% had a score of 3 or higher, indicating varying levels of comorbid conditions. Throughout the study period, there was a notable shift in the age distribution of those diagnosed with anaphylaxis. Specifically, the proportion of diagnosed children and adolescents, as well as young adults, gradually decreased, while the percentages of middle-aged and elderly groups increased, as illustrated in eFig. 1.

**Table 1:** The demographic and clinical characteristics.

Characteristics	Anaphylaxis^a^ (N = 37,012)	Single anaphylaxis^b^ (N = 31,229)	Recurrence of anaphylaxis^c^ (N = 5783)
Sex, N (%)			
Female	18,754 (50.67)	15,836 (50.71)	2918 (50.46)
Male	18,258 (49.33)	15,393 (49.29)	5865 (49.54)
Median age at index anaphylaixs^c^, years (IQR)	37 (20, 51)	36.0 (19, 51)	38 (19, 50)
Age group at index anaphylaixs^d^, N (%)			
Children and adolescents	11,158 (30.15)	9504 (30.43)	1654 (28.60)
Young adults	10,145 (27.41)	8559 (27.41)	1586 (27.43)
Middle-aged adults	11,586 (31.30)	9593 (30.72)	1993 (34.46)
Elderly	4123 (11.14)	3573 (11.44)	550 (9.51)
Season at index anaphylaxis^d^, N (%)			
Spring	8702 (23.51)	7350 (23.54)	1352 (23.38)
Summer	10,790 (29.15)	9136 (29.25)	1654 (28.60)
Autumn	10,477 (28.31)	8897 (28.49)	1580 (27.32)
Winter	7043 (19.03)	5846 (18.72)	1197 (20.70)
Calendar year at index anaphylaxis^d^, N (%)			
2002–2006	4384 (11.84)	3581 (11.47)	803 (13.89)
2007–2013	15,567 (42.06)	12,803 (41.00)	2764 (47.80)
2014–2019	17,061 (46.10)	14,845 (47.54)	2216 (38.32)
Neighborhood deprivation			
Low	9726 (27.37)	8308 (27.73)	1418 (25.44)
Intermediate	17,051 (47.99)	14,406 (48.09)	2645 (47.45)
High	8755 (24.64)	7244 (24.18)	1511 (27.11)
Economic status			
Low	5223 (14.60)	4433 (14.67)	790 (14.22)
Medium	16,217 (45.33)	13,654 (45.18)	2563 (46.15)
High	14,335 (40.07)	12,134 (40.15)	2201 (39.63)
Allergic comorbidity			
Atopic dermatitis	8035 (21.71)	6645 (21.28)	1390 (24.04)
Allergic rhinitis	11,187 (30.23)	9325 (29.86)	1862 (32.20)
Asthma	7222 (19.51)	6014 (19.26)	1208 (20.89)

Abbreviations: N, number; IQR, interquartile range.

a This includes individuals diagnosed with anaphylaxis at least once. Anaphylaxis was defined as meeting at least one of the following criteria: (1) emergency room visits or hospitalizations with diagnostic codes for food-related anaphylactic reactions, drug or vaccine-induced anaphylactic reactions, or anaphylactic shock; (2) outpatient visits coded for acute bronchospasm, stridor, or hypotension, accompanied by cardiopulmonary resuscitation or prescription of epinephrine or diphenhydramine; (3) emergency room visits or hospitalizations coded for allergic urticaria, angioneurotic edema, or poisoning due to adverse effects or medication overdose, with a concurrent prescription of epinephrine or diphenhydramine.

b Single anaphylaxis includes individuals with only one event of anaphylaxis.

c Recurrence of anaphylaxis includes individuals diagnosed with ≥2 diagnoses of anaphylaxis.

d The index anaphylaxis refers to the date of the initial diagnosis of anaphylaxis.

### Trends in the prevalence of anaphylaxis depending on age groups and sexes from 2002 to 2019

**Fig. 2:**
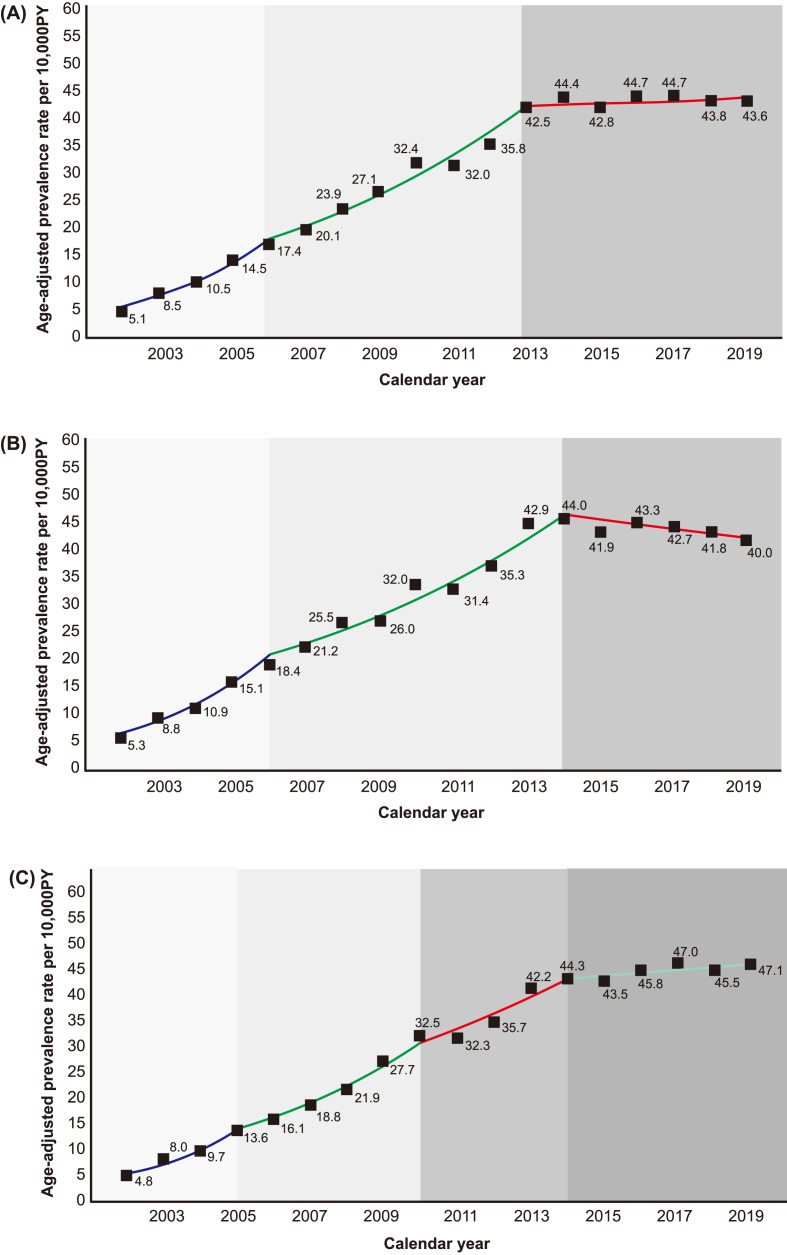
Age-adjusted prevalence of anaphylaxis by sex from 2002 to 2019, categorized as (A) overall, (B) male, and (C) female. Abbreviations: PY, person year. In each graph, the blue, green, red, and light blue solid lines represent the age-adjusted trend over the period. The background and solid color change with a significant shift in the prevalence trend. The filled squares with numbers indicate the age-adjusted prevalence of anaphylaxis per 10,000 person-years.

**Fig. 3:**
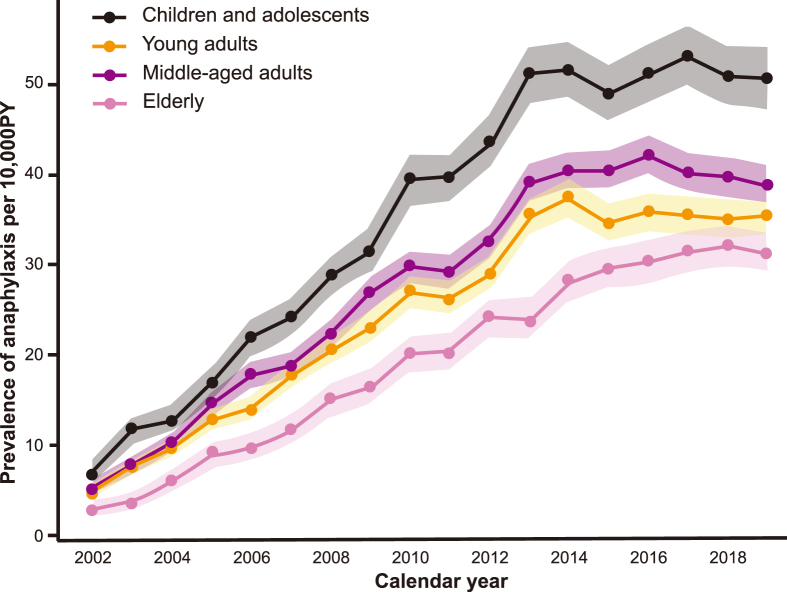
Prevalence of anaphylaxis from 2002 to 2019 by age groups. Abbreviations: PY, person year. A filled circle represents the prevalence of anaphylaxis per 10,000 person-years, while the shaded area indicates the standard error.

Our study observed a gradual increase in the prevalence of anaphylaxis from 2002 to 2019 across all age groups. The lowest incidence was recorded in 2002 (5.1 event/10,000 PY), and the highest incidence was observed in 2016 and 2017 (44.7 event/10,000 PY) (Fig. 2A–e Table 1). The age-adjusted prevalence initially surged from 2002 to 2006, then moderately rose until 2013, and stabilized thereafter. Both males and females exhibited similar prevalence patterns until 2014; however, from 2014 to 2019, a rising trend was noted in females, while a decrease was observed in males (Fig. 2B and C). Throughout the study period, children and adolescents consistently showed the highest prevalence of anaphylaxis, followed by middle-aged adults, young adults, and the elderly (Fig. 3). This pattern was observed in both males and females (eTables 2–3).

The trends in the prevalence of anaphylaxis from 2002 to 2019 are described in Table 2. Joinpoint regression analysis revealed 2 significant shifts in trends for the total population. There was a rapid increase in anaphylaxis prevalence (APC, 33.2; 95% CI, 24.29–59.57), followed by a gradual increase from 2006 to 2013 (APC, 12.75; 95% CI, 10.11–15.50), and a stable trend in 2013–2019 (APC, 0.61; 95% CI, −1.50–2.29). The elderly population demonstrated a prolonged increasing trend in prevalence up to 2015, with the highest AAPC (16.5; 95% CI, 15.1–20.6). Males had 2 joinpoints, whereas most female age groups had 3 joinpoints, except for children and adolescents. In males, the prevalence of anaphylaxis rapidly increased from 2002 to 2006 (APC, 34.4), exhibited a gradual increase from 2006 to 2014 (APC, 10.7), and stabilized thereafter (APC, −1.9). In females, a sharp increase was noted until 2005 (APC, 36.8), followed by a gradual rise until 2010 (APC, 18.3), and a steady trend from 2010 to 2019 (APC, 8.9 and 1.3). Overall, the AAPC over the study period was higher in females (APC, 13.7; 95% CI, 12.4–16.8) than in males (APC, 11.8; 95% CI, 10.7–14.2).

**Table 2:** Trends in anaphylaxis prevalence by age groups from 2002 to 2019.

Group^a^	Trend 1		Trend 2		Trend 3		Trend 4		AAPC^c^, % (95% CI) (2002–2019)
	APC^b^, % (95% CI)	Years	APC^b^, % (95% CI)	Years	APC^b^, % (95% CI)	Years	APC^b^, % (95% CI)	Years	
All sex									
All	33.15 (24.29–59.57)	2002–2006	12.75 (10.11–15.50)	2006–2013	0.61 (−1.50–2.29)	2013–2019			12.63 (11.60–15.33)
Children and adolescents	30.37 (23.39–42.28)	2002–2006	12.48 (9.58–15.28)	2006–2013	0.07 (−2.52–1.86)	2013–2019			11.75 (10.72–13.01)
Young adults	26.54 (20.62–42.55)	2002–2007	10.16 (7.29–13.46)	2007–2014	−1.23 (−6.36–1.60)	2014–2019			11.12 (9.93–13.36)
Middle aged adults	35.30 (26.94–51.55)	2002–2006	10.66 (8.76–12.97)	2006–2014	−1.09 (−4.34–1.24)	2014–2019			12.25 (11.19–14.05)
Elderly	54.63 (38.41–117.90)	2002–2005	17.33 (12.45–24.91)	2005–2010	8.94 (4.80–12.90)	2010–2015	1.57 (−4.13–4.51)	2015–2019	16.51 (15.14–20.62)
Male									
All	34.43 (25.29–56.51)	2002–2006	10.74 (8.81–12.92)	2006–2014	−1.94 (−5.07–0.36)	2014–2019			11.84 (10.72–14.16)
Children and adolescents	31.01 (24.13–45.17)	2002–2006	11.92 (9.41–14.62)	2006–2013	0.23 (−2.30–2.11)	2013–2019			11.71 (10.75–13.04)
Young adults	38.91 (23.75–81.29)	2002–2005	11.35 (8.17–14.32)	2005–2013	−2.11 (−6.44–0.52)	2013–2019			10.63 (9.24–13.40)
Middle aged adults	35.53 (25.17–66.52)	2002–2006	10.22 (7.79–13.06)	2006–2014	−3.03 (−8.06–0.01)>	2014–2019			11.43 (10.07–14.49)
Elderly	27.05 (17.91–85.13)	2002–2008	8.75 (6.69–15.74)	2008–2017	−7.95 (−18.19–4.55)	2017–2019			12.65 (10.77–19.01)
Female									
All	36.75 (22.92–78.83)	2002–2005	18.25 (9.61–23.91)	2005–2010	8.91 (−1.97–14.03)	2010–2014	1.26 (−4.87–6.34)	2014–2019	13.69 (12.43–16.80)
Children and adolescents	21.55 (8.71–56.03)	2002–2009	9.94 (−4.96–31.27)	2009–2013	0.58 (−11.58–8.31)	2013–2019			11.04 (9.52–14.21)
Young adults	46.72 (28.52–61.27)	2002–2004	19.97 (13.72–24.29)	2004–2009	9.54 (4.60–12.72)	2009–2014	1.04 (−2.39–2.93)	2014–2019	13.71 (12.48–14.81)
Middle aged adults	41.30 (30.47–70.09)	2002–2005	16.14 (10.29–23.10)	2005–2009	9.78 (−1.96–12.94)	2009–2014	1.13 (−3.65–5.79)	2014–2019	13.53 (12.53–15.63)
Elderly	83.42 (45.74–130.03)	2002–2004	22.86 (17.33–29.16)	2004–2009	9.85 (6.44–13.68)	2009–2014	3.20 (−0.55–5.01)	2014–2019	18.39 (16.47–21.44)

Abbreviations: APC, annual percentage change; CI, confidence interval; AAPC, average annual percentage change.

a Study population was categorized into four age brackets: a group of children and adolescents under 20 years, a group of young adults aged 20 to under 40 years, a group of middle aged adults aged 40 to under 60 years, and a group of elderly individuals aged 60 years and above.

b APC was calculated using joinpoint regression, which identifies significant changes in trends during the study period from 2002 to 2019.

c The AAPC represents a single measure of the average annual rate of change throughout the study period from 2002 to 2019.

### Risk for anaphylaxis recurrence

Out of the 37,012 individuals diagnosed with anaphylaxis, 5783 (15.6%) experienced recurring episodes during the study period (Table 1). The median age at the initial diagnosis of anaphylaxis was slightly higher for those with recurring episodes (38 years; IQR, 19–50 years) compared to those with a single episode (36 years; IQR, 19–51 years). Among those with recurring anaphylaxis, the median time between the first and second events was 10 months (IQR, 1–36 months), with about a quarter experiencing a second event within a month and another quarter being diagnosed between 1 month and 1 year after the first event. When comparing the seasons for index anaphylaxis between the 2 groups (single episode vs recurrent episodes), the proportion of patients experiencing anaphylaxis in winter was higher in the recurrent group compared to the single-episode group. However, the overall proportion of anaphylactic patients was higher in summer in both groups. Additionally, the proportion of individuals with high neighborhood deprivation or allergy comorbidities was higher in cases of recurrent anaphylaxis compared to single occurrences of anaphylaxis.

**Fig. 4:**
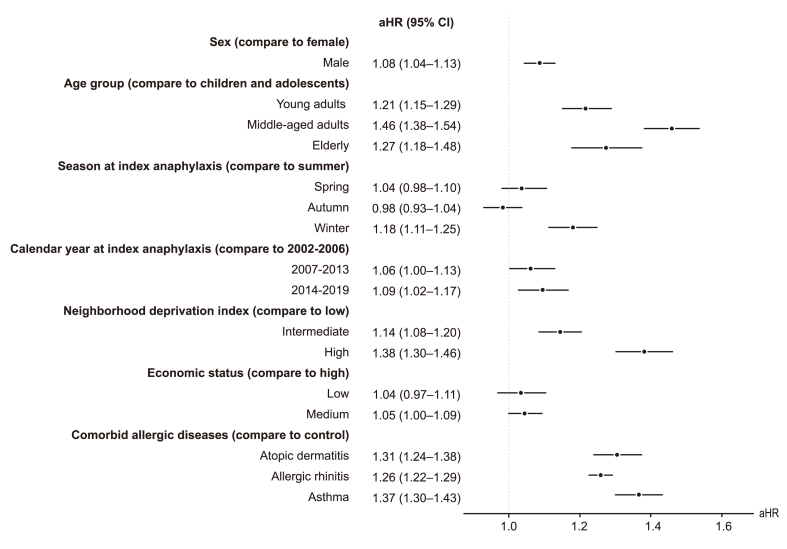
Demographic and clinical risk factors associated with the recurrence of anaphylaxis. The hazard ratio was calculated for the risk of recurrent anaphylaxis episodes for each characteristic and compared to the reference group by tracking the person-years for both single and recurrent anaphylaxis episodes within each group. The filled circle indicates the adjusted hazard ratio for the recurrence of anaphylaxis, and the black line indicates the corresponding 95% confidence interval. All analyses were adjusted for sex, age, and calendar year at the time of the initial anaphylaxis event, with the independent variable excluded from these adjustments. The Charlson Comorbidity Score was used to assess chronic conditions, including hypertension, diabetes, hyperlipidemia, osteoporosis, and chronic renal failure, using ICD-10 codes.

We assessed the risk factors for the recurrence of anaphylaxis, including sex, age, seasons, and calendar year at the index anaphylaxis, neighborhood deprivation index, and comorbidities (Fig. 4). Males exhibited a higher risk of recurrence compared to females (adjusted HR [aHR], 1.08; 95% CI, 1.04–1.13). The adult age group demonstrated a 1.2 to 1.5 times higher risk of recurrence than children and adolescents. The hazard ratio for anaphylaxis was higher in winter for the recurrent anaphylaxis group compared to the single anaphylaxis group, relative to summer (aHR = 1.18; 95% CI = 1.11–1.25). Furthermore, individuals residing in areas with intermediate (aHR, 1.14; 95% CI, 1.08–1.20) and high (aHR, 1.38; 95% CI, 1.30–1.46) neighborhood deprivation indices faced a significantly greater risk of recurrent anaphylaxis compared to those in areas with low deprivation. However, economic status, independent of neighborhood deprivation, did not significantly influence the risk of recurrence. The presence of allergic comorbidities, including atopic dermatitis, allergic rhinitis, and asthma, was associated with a 1.26 to 1.37 times increased risk of anaphylaxis recurrence. These findings remained consistent when considering death as a competing risk.

We analyzed the risk of anaphylaxis recurrence by age group (eFig. 2). In children and adolescents, there was no significant difference in anaphylaxis recurrence based on sex. In adults, the recurrence risk factors were similar to those observed in the overall age groups. Notably, middle-aged adults had a higher risk of recurrence compared to young adults, and a higher CCI score was associated with an increased recurrence risk.

## Discussion

In the present study, we reported national temporal trend of anaphylaxis prevalence across all ages and subgroups classified by sex and age. Additionally, we identified risk factors for recurrent anaphylaxis. Furthermore, anaphylaxis was defined using ICD-10 diagnosis codes in outpatient, emergency department, and inpatient settings,^14^ consistent with the definition reported by the World Allergy Organization.^19^ We observed a rapid increasing trend of anaphylaxis from 2002 to 2006, followed by gradual increases until 2013, and a steady state thereafter. This pattern was consistent across all age groups and both sexes, although there were variations in the rate of increase. Children and adolescents consistently showed the highest prevalence, while the elderly group experienced the largest rate of increase. Key risk factors for recurrent anaphylaxis included neighborhood deprivation status, not economic status, and the presence of comorbidities. Our study suggests that efforts to reduce the disease burden of anaphylaxis should focus on prevention strategies, particularly in areas of high neighborhood deprivation and comorbidities.

The strength of our study lies in its long-term analysis over 18 years and its comprehensive examination of anaphylaxis prevalence across all age groups. To the best of our knowledge, this study encompasses the longest study duration of epidemiological research on anaphylaxis since the 2000s, especially covering all age groups. In the epidemiologic studies of anaphylaxis, identification of definite cases without missing any instances is challenging, and the lack of a universally accepted, validated definition of anaphylaxis leads to variability in results across of study.^20^ By applying the validated case definition of anaphylaxis,^14^ we enhanced the precision of our data on anaphylaxis prevalence. In addition, we investigate a wide array of factors potentially related to recurrent anaphylaxis. This study is the first study which showed CCI is a risk factor for recurrent anaphylaxis in adults.

The reported prevalence of anaphylaxis varies based on factors such as medical utilization behavior, geographic regions, age of the population studies, and data source.^20^^,^^21^ In generally, studies on the prevalence of the anaphylaxis hospitalization has consistently shown increasing trends in the 1990s and 2000s, whether using either a nationwide population-based cohort or ICD-10 codes only.^2^, ^3^, ^4^ Even in studies investigating the anaphylaxis prevalence outside hospitalization, the prevalence showed an increasing trend from the early 2000s to the early 2010s, followed by a decline or stable patterns after the early 2010s.^5^^,^^6^ Our study corroborates these findings, showing a rising trend in anaphylaxis prevalence until mid-2010s, followed by a stationary or slightly decreasing trend thereafter in Korea. The increasing trend of recurrence rate over time might be attributable to a greater exposure to causative factors and increased awareness and recognition of anaphylaxis by trained physicians, care-givers, and patients.^22^

The recurrence rate of anaphylaxis varies between studies, with higher rate noted in studies with longer follow-up period: 10.6% in the 12-month after the index anaphylaxis date,^8^ 8% at a mean of 1.8 years follow-up after the index episode of anaphylaxis,^7^ and 26.5–54.0% during a follow-up of 1.5–25 years.^20^ In our study, the recurrence rate of anaphylaxis was 15.6%, which might be the relatively low considering the extended follow-up period. Variation in the causes of anaphylaxis, regional differences in education levels on the prevention measures, and the definition of anaphylaxis used in each study could influence the recurrence rate.

To enhance public health related to anaphylaxis, several studies have pinpointed risk factors of recurrent anaphylaxis. In our study, we found that comorbid diseases and neighborhood deprivation are strong risk factors of recurrent anaphylaxis across all age groups, including both adult and children groups. Previous studies reported that history of atopic dermatitis^7^ or comorbidities of allergic rhinitis and asthma^8^ heightened the risk of recurrent anaphylaxis. The findings emphasize the importance of being vigilant about the high risk of recurrent anaphylaxis in patients with allergic comorbid conditions. Adults with a high CCI score are at higher risk for anaphylaxis recurrence, which could be explained by several factors. Their compromised health status from multiple comorbidities could lead to immune system dysfunction or systemic inflammation, increasing the severity and frequency of anaphylactic episodes.^23^ Additionally, several drugs used to treat comorbidities, such as aspirin, angiotensin-converting enzyme inhibitors, or beta-blockers, could decrease the threshold of anaphylaxis reaction.^24^^,^^25^ Only few studies have reported the association of socioeconomic status with anaphylaxis, yielding inconclusive results.^26^^,^^27^ Our study identified that high neighborhood deprivation, rather than income levels, increased the risk of recurrent anaphylaxis. This suggest that living in an undeserved area may be associated with increased healthcare utilization for recurrent anaphylaxis, potentially due to inadequate health literacy, lifestyle factors, and increased exposure to culprit allergens. The high neighborhood deprivation could also be linked with poor accessibility to appropriate medical systems and insufficient education on the preventive strategies for anaphylaxis. Therefore, early recognition and diagnosis of anaphylaxis, along with proper education on its management at the time of the initial event, are crucial to reduce the disease burden, especially in areas of high neighborhood deprivation.

Nonetheless, there are some limitations. Despite our use of national health insurance system data to comprehensively cover all ages for long-term epidemiology of anaphylaxis, the NHIS-NSC data system has limitations in defining the anaphylaxis cases using ICD-10 codes, which have inherent limitations in sensitivity and specificity of anaphylaxis diagnosis.^28^^,^^29^ Validation of other data is required for the robustness of our study results. The data system also has limitations in identifying the trigger of each anaphylaxis episode and anaphylaxis severity, which could be associated with the cause and prediction of recurrence. Additionally, the detailed clinical characteristics of each individual subject are not fully reflected in this data set. However, the NHIS in South Korea provides uniform health care benefits, including diagnosis, prescriptions, hospitalizations, under both Medical Aid and National Health Insurance.

In conclusion, we found a longitudinal trend of anaphylaxis from 2002 to 2019, characterized by a rapid increase in 2006, a subsequent gradual increase until 2013, and then a stable trend in 2013–2019. Children and adolescents had the highest prevalence, whereas the elderly exhibited the highest increase rate. Factors such as sex, age groups, seasons during the first anaphylaxis episode, comorbid allergic diseases, CCI score, and neighborhood deprivation could increase the risk of recurrent anaphylaxis. Thus, these data can help establish public health policy to decrease the disease burden in the future.

## Authors’ consent for publication

All authors have read and approved the final version of the manuscript and agree with its submission to *World Allergy Organization Journal*.

## Data availability

This study was based on the National Health Claims Database (NHIS-2019-1-560) established by the NHIS of the Republic of Korea. Applications using NHIS data are reviewed by the Inquiry Committee of Research Support, and if the application is approved, raw data are provided to the applicant for a fee. We cannot provide access to the data, analytical methods, and research materials to other researchers because of the intellectual property rights of this database, which is owned by the National Health Insurance Corporation. However, investigators who wish to reproduce our results or replicate the procedure can use the database, which is open for research purposes (https://nhiss.nhis.or.kr/).

## Author contributions

Full access to all of the data in the study and responsibility for the integrity of the data and the accuracy of the data analysis: Man Yong Han and Eun Lee.

Concept and design: Ju Hee Kim, Man Yong Han, and Eun Lee.

Acquisition, analysis, or interpretation of data: Man Yong Han, Nahyun Lee, and Bo Eun Han.

Drafting of the manuscript: Eun Lee and Ju Hee Kim.

Critical revision of the manuscript for important intellectual content: Eun Kyo Ha and Jeewon Shin.

Obtained funding: Man Yong Han and Eun Lee.

Administrative, technical, or material support: Nahyun Lee and Bo Eun Han.

Supervision: Man Yong Han and Eun Lee.

## Statement of ethics

This study was conducted with ethical approval from the current National Health Insurance Act. The use of de-identified individual data for research purposes was authorized under the National Health Insurance Act. The study protocol was reviewed and approved by the institutional review board of the Korea National Institute for Bioethics Policy (P01-201603-21-005).

## Funding

This work was supported by a grant of the Korea Health Technology R&D Project through the Korea Health Industry Development Institute (KHIDI), funded by the Ministry of Health & Welfare, Republic of Korea (grant number: HR22C1605) and by the National Research Foundation of Korea (NRF) grant funded by the Korea government (MSIT) (NRF-2022R1A2C2011078). The funders had no role in the design and conduct of the study; collection, management, analysis, and interpretation of the data; preparation, review, or approval of the manuscript; and decision to submit the manuscript for publication.

## Declaration of competing interest

The authors declare that this study was conducted in the absence of any commercial or financial relationships that could be interpreted as potential conflicts of interest.
