# Supplementary material for: National trends in the prevalence and recurrence of anaphylaxis across all ages: The role of neighborhood deprivation and comorbidity (2002–2019)
Source: World Allergy Organ J. 2024 Dec 2;17(12):101005. doi: 10.1016/j.waojou.2024.101005 (PMC11652768; doi:10.1016/j.waojou.2024.101005)
Supplement: Multimedia component 1 [file mmc1.doc]

eFigure 1. Distribution of participants throughout the study period by age groups.


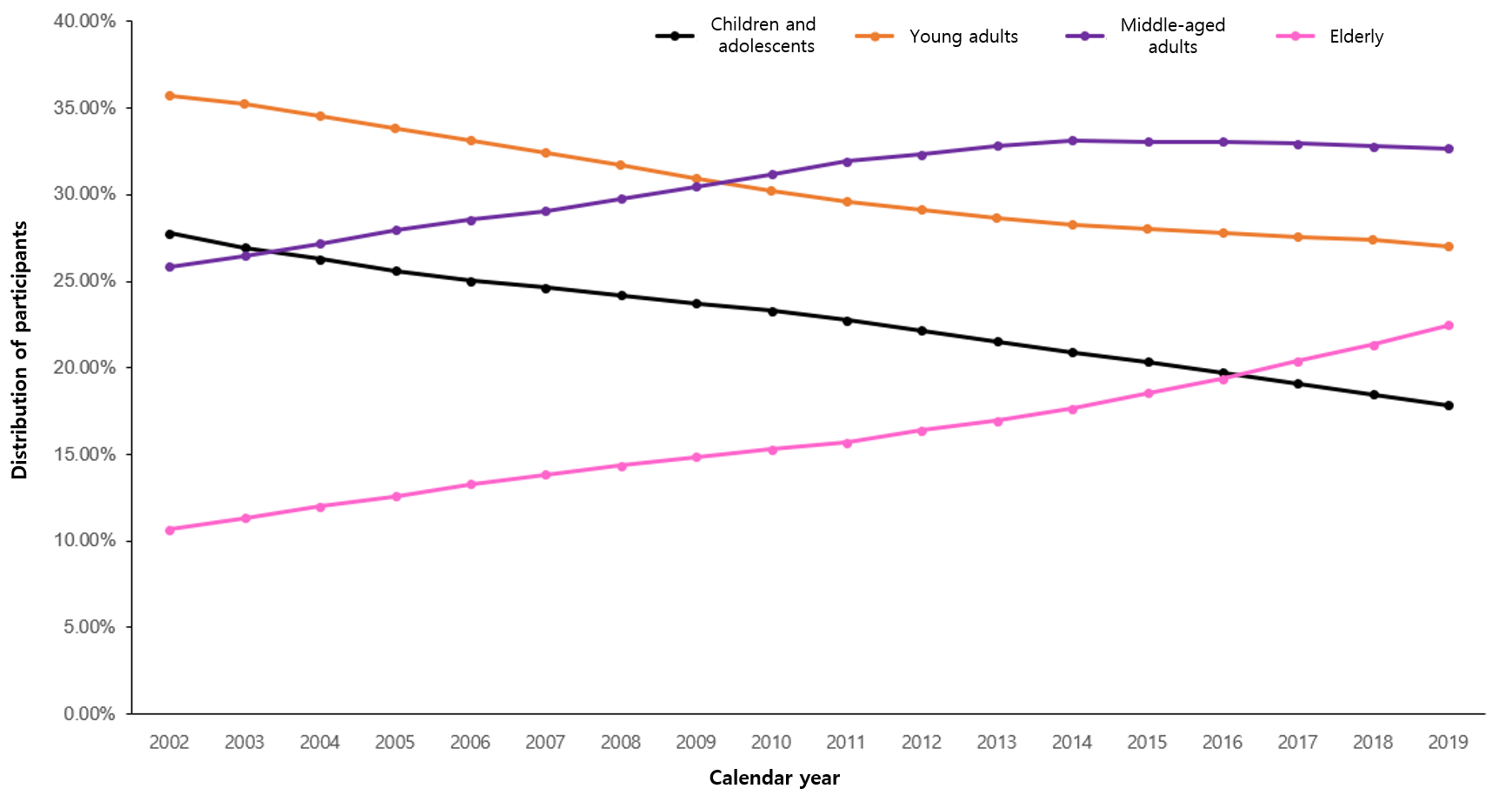


eFigure 2. The risk for the recurrence of anaphylaxis in (A) children and adolescents and (B) adults.


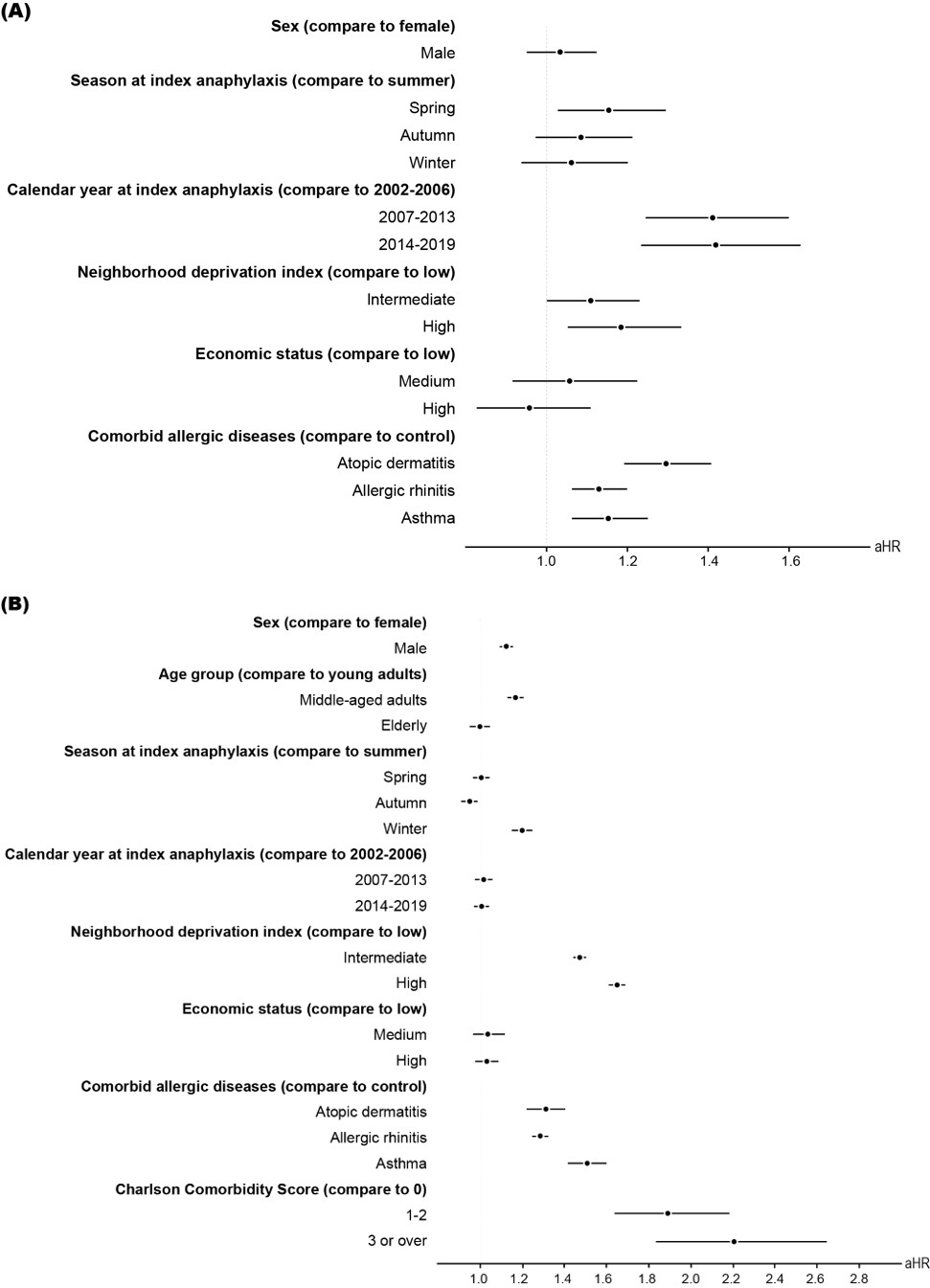


A filled round indicates the adjusted hazard ratio for the recurrence of anaphylaxis, while the black line indicates the corresponding 95% confidence intervals. All analyses were adjusted for sex, age groups, and calendar years at the time of the initial anaphylaxis event, with the independent variable excluded from these adjustments.

The Charlson Comorbidity Score was used to assess chronic conditions, including hypertension (HTN), diabetes (DM), hyperlipidemia (HPL), osteoporosis (OP), and chronic renal failure, using ICD-10 codes.

eTable 1. Prevalence of anaphylaxis by age groups.

| Year | All ages | | | | Children and adolescents | | | Young adult | | | Middle-aged adult | | | Elderly | | |
| --- | --- | --- | --- | --- | --- | --- | --- | --- | --- | --- | --- | --- | --- | --- | --- | --- |
|  | N | event^1^ | IR  /10,000PY^1^ | SE | N | event | IR  /10,000PY | N | event | IR  /10,000PY | N | event | IR  /10,000PY | N | event | IR  /10,000PY |
| 2002 | 982,451 | 499 | 5.1 | 0.022 | 272,750 | 182 | 6.7 | 350,785 | 162 | 4.6 | 254,102 | 130 | 5.1 | 104,814 | 25 | 2.4 |
| 2003 | 992,833 | 836.2 | 8.5 | 0.028 | 267,609 | 319 | 11.9 | 349,860 | 270 | 7.7 | 262,852 | 202 | 7.7 | 112,512 | 39 | 3.5 |
| 2004 | 1,002,496 | 1027.8 | 10.5 | 0.031 | 263,444 | 336 | 12.8 | 346,286 | 334 | 9.7 | 272,540 | 277 | 10.2 | 120,226 | 72 | 6.0 |
| 2005 | 1,011,638 | 1425 | 14.5 | 0.035 | 259,057 | 436 | 16.8 | 342,147 | 443 | 13.0 | 282,938 | 416 | 14.7 | 127,496 | 118 | 9.3 |
| 2006 | 1,021,201 | 1712.5 | 17.4 | 0.038 | 255,527 | 563 | 22.0 | 338,270 | 474 | 14.0 | 291,699 | 521 | 17.9 | 135,705 | 131 | 9.7 |
| 2007 | 1,026,676 | 1972.9 | 20.1 | 0.040 | 253,048 | 614 | 24.3 | 333,150 | 596 | 17.9 | 298,502 | 560 | 18.8 | 141,976 | 165 | 11.6 |
| 2008 | 1,031,321 | 2350.3 | 23.9 | 0.042 | 249,635 | 723 | 29.0 | 326,749 | 673 | 20.6 | 306,987 | 686 | 22.4 | 147,950 | 224 | 15.1 |
| 2009 | 1,035,500 | 2665 | 27.1 | 0.044 | 245,909 | 778 | 31.6 | 320,206 | 739 | 23.1 | 315,590 | 849 | 26.9 | 153,795 | 253 | 16.5 |
| 2010 | 1,040,209 | 3180.4 | 32.4 | 0.046 | 242,429 | 959 | 39.6 | 314,326 | 855 | 27.2 | 324,247 | 969 | 29.9 | 159,207 | 321 | 20.2 |
| 2011 | 1,044,798 | 3140.2 | 32.0 | 0.046 | 237,745 | 941 | 39.6 | 309,253 | 812 | 26.3 | 333,705 | 972 | 29.1 | 164,095 | 332 | 20.2 |
| 2012 | 1,049,457 | 3519.1 | 35.8 | 0.047 | 232,667 | 1017 | 43.7 | 305,749 | 889 | 29.1 | 339,086 | 1105 | 32.6 | 171,955 | 417 | 24.3 |
| 2013 | 1,053,170 | 4179.8 | 42.5 | 0.048 | 226,854 | 1163 | 51.3 | 302,083 | 1078 | 35.7 | 345,796 | 1360 | 39.3 | 178,437 | 425 | 23.8 |
| 2014 | 1,056,855 | 4363 | 44.4 | 0.048 | 221,091 | 1144 | 51.7 | 298,997 | 1123 | 37.6 | 350,183 | 1420 | 40.6 | 186,584 | 528 | 28.3 |
| 2015 | 1,060,351 | 4203.8 | 42.8 | 0.048 | 215,756 | 1057 | 49.0 | 297,213 | 1031 | 34.7 | 350,734 | 1424 | 40.6 | 196,648 | 582 | 29.6 |
| 2016 | 1,063,251 | 4388.7 | 44.7 | 0.048 | 209,937 | 1079 | 51.4 | 295,669 | 1061 | 35.9 | 351,426 | 1488 | 42.3 | 206,219 | 627 | 30.4 |
| 2017 | 1,064,877 | 4392.7 | 44.7 | 0.048 | 203,312 | 1083 | 53.3 | 293,508 | 1041 | 35.5 | 351,003 | 1415 | 40.3 | 217,054 | 686 | 31.6 |
| 2018 | 1,065,646 | 4304.2 | 43.8 | 0.048 | 197,002 | 1004 | 51.0 | 291,651 | 1023 | 35.1 | 349,457 | 1391 | 39.8 | 227,536 | 730 | 32.1 |
| 2019 | 1,066,077 | 4286.4 | 43.6 | 0.048 | 190,411 | 967 | 50.8 | 287,567 | 1021 | 35.5 | 348,379 | 1359 | 39.0 | 239,720 | 751 | 31.3 |

Abbreviations: IR, incidence rate; PY, person year.

eTable 2. Prevalence of anaphylaxis by age groups in males.

| Year | All ages | | | | Children and adolescents | | | Young adult | | | Middle-aged adult | | | Elderly | | |
| --- | --- | --- | --- | --- | --- | --- | --- | --- | --- | --- | --- | --- | --- | --- | --- | --- |
|  | N | event^1^ | IR  /10,000PY^1^ | SE | N | event | IR  /10,000PY | N | event | IR  /10,000PY | N | event | IR  /10,000PY | N | event | IR  /10,000PY |
| 2002 | 491,660 | 262.0 | 5.3 | 0.032 | 143,194 | 101 | 7.1 | 179,058 | 84 | 4.7 | 127,245 | 64 | 5.0 | 42,163 | 13 | 3.1 |
| 2003 | 497,118 | 434.8 | 8.8 | 0.040 | 140,705 | 177 | 12.6 | 178,551 | 128 | 7.2 | 132,154 | 109 | 8.2 | 45,708 | 18 | 3.9 |
| 2004 | 502,087 | 534.0 | 10.9 | 0.044 | 138,491 | 207 | 14.9 | 176,851 | 150 | 8.5 | 137,364 | 140 | 10.2 | 49,381 | 32 | 6.5 |
| 2005 | 506,820 | 743.2 | 15.1 | 0.050 | 136,177 | 250 | 18.4 | 174,949 | 225 | 12.9 | 142,799 | 202 | 14.1 | 52,895 | 60 | 11.3 |
| 2006 | 511,758 | 905.9 | 18.4 | 0.054 | 134,255 | 327 | 24.4 | 172,859 | 231 | 13.4 | 147,625 | 282 | 19.1 | 57,019 | 53 | 9.3 |
| 2007 | 514,430 | 1042.8 | 21.2 | 0.057 | 132,911 | 374 | 28.1 | 170,611 | 289 | 16.9 | 150,794 | 281 | 18.6 | 60,114 | 76 | 12.6 |
| 2008 | 516,711 | 1254.4 | 25.5 | 0.061 | 131,058 | 426 | 32.5 | 167,632 | 329 | 19.6 | 154,893 | 370 | 23.9 | 63,128 | 109 | 17.3 |
| 2009 | 518,683 | 1278.5 | 26.0 | 0.061 | 128,951 | 423 | 32.8 | 164,607 | 317 | 19.3 | 159,154 | 417 | 26.2 | 65,971 | 100 | 15.2 |
| 2010 | 520,938 | 1573.0 | 32.0 | 0.065 | 126,827 | 542 | 42.7 | 161,919 | 371 | 22.9 | 163,650 | 476 | 29.1 | 68,542 | 150 | 21.9 |
| 2011 | 523,045 | 1545.6 | 31.4 | 0.064 | 124,291 | 523 | 42.1 | 159,425 | 354 | 22.2 | 168,493 | 482 | 28.6 | 70,836 | 149 | 21.0 |
| 2012 | 525,335 | 1733.2 | 35.3 | 0.066 | 121,503 | 565 | 46.5 | 157,803 | 399 | 25.3 | 171,277 | 538 | 31.4 | 74,752 | 184 | 24.6 |
| 2013 | 527,017 | 2107.2 | 42.9 | 0.068 | 118,221 | 659 | 55.7 | 156,334 | 488 | 31.2 | 174,616 | 697 | 39.9 | 77,846 | 186 | 23.9 |
| 2014 | 528,681 | 2161.7 | 44.0 | 0.068 | 114,868 | 640 | 55.7 | 155,155 | 509 | 32.8 | 176,803 | 696 | 39.4 | 81,855 | 241 | 29.4 |
| 2015 | 530,369 | 2058.1 | 41.9 | 0.068 | 111,899 | 615 | 55.0 | 154,549 | 430 | 27.8 | 177,165 | 685 | 38.7 | 86,756 | 269 | 31.0 |
| 2016 | 531,745 | 2128.4 | 43.3 | 0.068 | 108,665 | 614 | 56.5 | 154,066 | 443 | 28.8 | 177,539 | 721 | 40.6 | 91,475 | 276 | 30.2 |
| 2017 | 532,421 | 2098.8 | 42.7 | 0.068 | 105,228 | 591 | 56.2 | 153,181 | 428 | 27.9 | 177,170 | 670 | 37.8 | 96,842 | 350 | 36.1 |
| 2018 | 532,728 | 2053.3 | 41.8 | 0.068 | 101,812 | 565 | 55.5 | 152,366 | 406 | 26.6 | 176,514 | 668 | 37.8 | 102,036 | 343 | 33.6 |
| 2019 | 532,763 | 1967.5 | 40.0 | 0.067 | 98,289 | 537 | 54.6 | 150,454 | 411 | 27.3 | 176,045 | 579 | 32.9 | 107,975 | 322 | 29.8 |

Abbreviations: IR, incidence rate; PY, person year.

eTable 3. Prevalence of anaphylaxis by age groups in females.

| Year | All ages | | | | Children and adolescents | | | Young adult | | | Middle-aged adult | | | Elderly | | |
| --- | --- | --- | --- | --- | --- | --- | --- | --- | --- | --- | --- | --- | --- | --- | --- | --- |
|  | N | event^1^ | IR  /10,000PY^1^ | SE | N | event | IR  /10,000PY | N | event | IR  /10,000PY | N | event | IR  /10,000PY | N | event | IR  /10,000PY |
| 2002 | 490,791 | 237.0 | 4.8 | 0.031 | 129,556 | 81 | 6.3 | 171,727 | 78 | 4.5 | 126,857 | 66 | 5.2 | 62,651 | 12 | 1.9 |
| 2003 | 495,715 | 392.7 | 8.0 | 0.039 | 126,904 | 142 | 11.2 | 171,309 | 142 | 8.3 | 130,698 | 93 | 7.1 | 66,804 | 21 | 3.1 |
| 2004 | 500,409 | 476.5 | 9.7 | 0.042 | 124,953 | 129 | 10.3 | 169,435 | 184 | 10.9 | 135,176 | 137 | 10.1 | 70,845 | 40 | 5.6 |
| 2005 | 504,818 | 665.8 | 13.6 | 0.048 | 122,880 | 186 | 15.1 | 167,198 | 218 | 13.0 | 140,139 | 214 | 15.3 | 74,601 | 58 | 7.8 |
| 2006 | 509,443 | 789.9 | 16.1 | 0.051 | 121,272 | 236 | 19.5 | 165,411 | 243 | 14.7 | 144,074 | 239 | 16.6 | 78,686 | 78 | 9.9 |
| 2007 | 512,246 | 921.1 | 18.8 | 0.055 | 120,137 | 240 | 20.0 | 162,539 | 307 | 18.9 | 147,708 | 279 | 18.9 | 81,862 | 89 | 10.9 |
| 2008 | 514,610 | 1074.8 | 21.9 | 0.058 | 118,577 | 297 | 25.0 | 159,117 | 344 | 21.6 | 152,094 | 316 | 20.8 | 84,822 | 115 | 13.6 |
| 2009 | 516,817 | 1359.8 | 27.7 | 0.062 | 116,958 | 355 | 30.4 | 155,599 | 422 | 27.1 | 156,436 | 432 | 27.6 | 87,824 | 153 | 17.4 |
| 2010 | 519,271 | 1595.3 | 32.5 | 0.065 | 115,602 | 417 | 36.1 | 152,407 | 484 | 31.8 | 160,597 | 493 | 30.7 | 90,665 | 171 | 18.9 |
| 2011 | 521,753 | 1587.6 | 32.3 | 0.065 | 113,454 | 418 | 36.8 | 149,828 | 458 | 30.6 | 165,212 | 490 | 29.7 | 93,259 | 183 | 19.6 |
| 2012 | 524,122 | 1753.6 | 35.7 | 0.066 | 111,164 | 452 | 40.7 | 147,946 | 490 | 33.1 | 167,809 | 567 | 33.8 | 97,203 | 233 | 24.0 |
| 2013 | 526,153 | 2071.9 | 42.2 | 0.068 | 108,633 | 504 | 46.4 | 145,749 | 590 | 40.5 | 171,180 | 663 | 38.7 | 100,591 | 239 | 23.8 |
| 2014 | 528,174 | 2174.3 | 44.3 | 0.068 | 106,223 | 504 | 47.4 | 143,842 | 614 | 42.7 | 173,380 | 724 | 41.8 | 104,729 | 287 | 27.4 |
| 2015 | 529,982 | 2136.5 | 43.5 | 0.068 | 103,857 | 442 | 42.6 | 142,664 | 601 | 42.1 | 173,569 | 739 | 42.6 | 109,892 | 313 | 28.5 |
| 2016 | 531,506 | 2246.9 | 45.8 | 0.068 | 101,272 | 465 | 45.9 | 141,603 | 618 | 43.6 | 173,887 | 767 | 44.1 | 114,744 | 351 | 30.6 |
| 2017 | 532,456 | 2307.2 | 47.0 | 0.068 | 98,084 | 492 | 50.2 | 140,327 | 613 | 43.7 | 173,833 | 745 | 42.9 | 120,212 | 336 | 28.0 |
| 2018 | 532,918 | 2232.8 | 45.5 | 0.068 | 95,190 | 439 | 46.1 | 139,285 | 617 | 44.3 | 172,943 | 723 | 41.8 | 125,500 | 387 | 30.8 |
| 2019 | 533,314 | 2311.2 | 47.1 | 0.068 | 92,122 | 430 | 46.7 | 137,113 | 610 | 44.5 | 172,334 | 780 | 45.3 | 131,745 | 429 | 32.6 |

Abbreviations: IR, incidence rate; PY, person year.
